# An interpretable computational phenotyping pipeline for adult autism using routine electronic health records

**DOI:** 10.3389/fpsyt.2026.1952612

**Published:** 2026-09-15

**Authors:** Jordi Rodeiro-Boliart, Maria Nuñez-Jimenez, Beatriz Olaya, Elena Huerta-Ramos, Elisabet Golobardes

**Affiliations:** 1 Impacte i prevenció dels trastorns mentals, Institut de Recerca Sant Joan de Déu, Sant Boi de Llobregat, Spain; 2 Smart Society Research Group, Engineering Department, La Salle-Universitat Ramon Llull, Barcelona, Spain; 3 Department of Psychiatry and Neurochemistry, Institute of Neuroscience and Physiology at the Sahlgrenska Academy, University of, Gothenburg, Sweden; 4 Etiopathogenesis and Treatment of Severe Mental Disorders, Sant Joan de Déu Research Institute, Sant Joan de Déu Foundation, Barcelona, Spain; 5 Biomedical Research in the Mental Health Network (CIBERSAM), Carlos III Health Institute, Madrid, Spain; 6 Department of Clinical and Health Psychology, Autonomous University of Barcelona, Bellaterra, Spain

**Keywords:** autism spectrum disorder, clustering, computational phenotyping, electronic health records, explainable AI, mental health, self-organizing map

## Abstract

**Introduction:**

Electronic health records (EHRs) provide new opportunities for computational phenotyping in mental health, but routinely collected diagnostic data are often sparse, heterogeneous, and challenging to interpret. While numerous computational approaches have been applied to identify patient subgroups, clinically useful phenotyping requires workflows capable of transforming routine diagnostic information into interpretable patient profiles. The goal of this work is to develop and evaluate an end-to-end computational phenotyping pipeline for routine ICD-10 diagnostic data and demonstrate its feasibility in adults with autism spectrum disorder (ASD).

**Methods:**

A retrospective observational study was conducted using routine EHR data from adults with ASD receiving care at a specialized mental health service. ICD-10 diagnoses were extracted, aggregated into clinically meaningful diagnostic categories, and transformed into binary patient-level representations. A self-organizing map (SOM) was used to organize patients according to diagnostic similarity, followed by hierarchical clustering to identify phenotype groups. Cluster solutions were evaluated using internal validity metrics, cluster size distributions, and interpretability. The resulting phenotypes were identified through diagnostic prevalence profiles and SOM-based visualizations.

**Results:**

The study included 927 adults with ASD, of whom 744 presented at least one comorbid diagnostic category and were included in the computational phenotyping analysis. The proposed pipeline identified four phenotypes characterized by distinct patterns of psychiatric and developmental comorbidity. The SOM structure provided an interpretable visualization of the diagnostic landscape, while hierarchical clustering enabled the identification of coherent phenotype groups. Internal validation metrics supported the selected solution while preserving informative subgroup sizes.

**Discussion:**

This study presents an end-to-end computational phenotyping pipeline that transforms routinely collected psychiatric EHR data into interpretable patient phenotypes. By integrating diagnostic extraction, clinically informed aggregation, representation learning, clustering, visualization, and characterization, the workflow provides a practical framework for analyzing complex diagnostic data in real-world mental health settings. Future studies should evaluate its applicability across other psychiatric and neurodevelopmental populations.

## 1 Introduction

The increasing availability of electronic health records (EHRs) has created new opportunities to characterize clinical heterogeneity using routinely collected healthcare data (1). In mental health, where patients frequently present with multiple co-occurring conditions, EHRs provide a valuable source of information on diagnostic patterns, disease burden, and healthcare trajectories. Computational phenotyping approaches seek to transform these routinely collected clinical data into structured patient profiles that facilitate the identification of clinically meaningful subgroups and sources of heterogeneity (2, 3).

Despite their potential, routine diagnostic records present important challenges for computational analysis. Diagnostic information based on the International Classification of Diseases (ICD) (4) was originally developed for clinical documentation, epidemiological reporting, and administrative purposes rather than machine learning applications. Consequently, diagnostic datasets are often sparse, high-dimensional, and characterized by heterogeneous levels of granularity (5, 6). Furthermore, similarities between patient profiles are not always adequately captured by the hierarchical structure of diagnostic classifications, complicating the identification of meaningful patterns within large patient populations.

To address these challenges, a variety of computational phenotyping approaches have been applied to EHR data. While important advances have been achieved using supervised approaches based on predefined distinctions, increasing attention has been directed toward unsupervised methods capable of identifying previously unrecognized multivariate patterns (7). These include hierarchical clustering, latent class methods, tensor factorization techniques, and natural language processing (NLP)-based phenotype discovery (2, 8). These methods have demonstrated the potential of EHRs for identifying clinically relevant patient subgroups and advancing precision medicine. However, many approaches primarily focus on subgroup identification while providing limited insight into how patient groups relate to one another or how the resulting phenotypes can be interpreted by clinicians. In psychiatric research, where clinical adoption depends heavily on transparency and interpretability, there remains a need for computational phenotyping frameworks capable of transforming routine diagnostic information into clinically understandable representations of patient heterogeneity (9, 10).

Such frameworks should not only identify groups of patients with similar clinical characteristics but also provide an interpretable structure of the underlying diagnostic landscape, as the lack of interpretability remains a barrier to the translation of computational methods into routine mental health practice (10, 11). Visualization of patient similarities may facilitate exploration of continuous patterns of heterogeneity, inspection of relationships between phenotypes, and communication of findings to clinical experts (12, 13). Consequently, there is a need for end-to-end workflows that integrate data preprocessing, patient representation, phenotype identification, visualization, and clinical interpretation within a single reproducible pipeline.

To address this need, we developed a computational phenotyping pipeline for routine psychiatric EHR data. The proposed workflow combines clinically informed aggregation of ICD-10 diagnoses, construction of patient-level diagnostic profiles, topology-preserving organization of patients using a Self-Organizing Map (SOM) (14), phenotype identification through clustering, visualization of diagnostic patterns, and expert clinical interpretation of the resulting phenotypes. Within this framework, the SOM acts as an interpretable representation-learning component that preserves relationships between similar patient profiles while supporting visualization and exploration of complex diagnostic structures (15). SOM-based approaches have previously demonstrated utility for the analysis and visualization of high-dimensional healthcare data, making them particularly suitable for exploratory phenotyping tasks (16).

Adult autism spectrum disorder (ASD) provides a relevant context in which to evaluate such an approach. Adults with ASD frequently present diverse combinations of psychiatric and neurodevelopmental comorbidities, resulting in substantial clinical heterogeneity. Autism research has increasingly emphasized the importance of complementing supervised analyses based on predefined distinctions with unsupervised approaches capable of uncovering previously unrecognized patterns within heterogeneous populations. A more refined understanding of this diversity may ultimately support precision-oriented research and more individualized clinical care (7, 17). Previous EHR-based studies have demonstrated the existence of distinct patterns of co-occurring conditions within autistic populations, highlighting both the complexity of these patients and the need for methods capable of generating clinically interpretable structures of this heterogeneity (18).

The objective of this study was to develop and evaluate an interpretable computational phenotyping pipeline capable of transforming routinely collected ICD-10 diagnostic information into clinically meaningful patient phenotypes. Using adults with ASD as a real-world use case, we assessed whether routine psychiatric EHR data could be organized into coherent and interpretable phenotypic representations through the integration of diagnostic preprocessing, representation learning, clustering, visualization, and expert clinical review.

The remainder of this article is organized as follows. First, we describe the study population and the computational phenotyping workflow, including diagnostic preprocessing, patient representation, and phenotype generation procedures. We then present the resulting phenotype structure and robustness analyses. Finally, we discuss the methodological implications, strengths, limitations, and potential applications of the proposed framework.

## 2 Methods

### 2.1 Study design and population

We conducted a retrospective observational study using EHR data from Parc Sanitari Sant Joan de Déu in Sant Boi de Llobregat, Barcelona (Spain). The study population included adults (≥18 years) with a clinician-assigned ASD diagnosis (ICD-10 F84.x) who attended the service between May 9, 2017 and July 10, 2025.

This study was conducted in accordance with the ethical principles of the Declaration of Helsinki and current data protection regulations (EU GDPR 2016/679). The research protocol titled “*Leveraging Artificial Intelligence for Enhanced Screening and Diagnosis of Autism Spectrum Disorder in Adults through Medical Records Analysis*” was reviewed and approved by the Comité de Ética de la Investigación con Medicamentos (CEIm) of Parc Sanitari Sant Joan de Déu (Barcelona, Spain), under approval code PIC-194-24. The study involved the retrospective analysis of fully anonymized hospital data; therefore, the requirement for informed consent was waived in accordance with institutional and national ethical guidelines. Data anonymization was ensured from the outset, with no identifiable or re-identifiable information present, complying with current data protection standards.

Because the analysis was based exclusively on the presence or absence of aggregated diagnostic categories, the anonymization process did not remove information required for phenotype generation or alter the structure of the diagnostic representations used in the study.

### 2.2 Computational phenotyping pipeline

The analysis consisted of five consecutive steps (1): Diagnostic data extraction and preprocessing, (2) Construction of patient-level diagnostic representations, (3) Topology-preserving organization of patients using a Self-Organizing Map (4) Identification of phenotype groups through hierarchical clustering, and (5) Visualization and characterization of the resulting phenotypes (6) Clinical review of phenotype plausibility and interpretation.

Analyses were conducted in Python 3.11. Data manipulation was performed using pandas (v2.3.3) and NumPy (v1.23.5). Visualizations were generated using Matplotlib (v3.10.7) and Seaborn (v0.13.2). Hierarchical clustering analyses were performed using SciPy (v1.15.3). Self-Organizing Maps were implemented using MiniSom (v2.3.6).

### 2.3 Diagnostic data extraction and preprocessing

Diagnostic information was extracted from routinely recorded ICD-10 diagnoses. Diagnoses from Chapter F and selected neurodevelopmentally relevant diagnoses from Chapters G and Q were included.

To reduce sparsity while preserving clinical meaning, diagnoses were aggregated according to the hierarchical organization of ICD-10. Specifically, diagnoses were grouped at the block level within chapters, preserving clinically recognized diagnostic entities while avoiding excessive fragmentation associated with individual ICD-10 codes. Broader chapter-level aggregation was considered insufficiently specific for meaningful clinical interpretation.

Diagnostic categories with a prevalence below 1.5% of the cohort were excluded during preprocessing. In the study sample (N = 927), this corresponded to fewer than 14 individuals. This filtering step was applied to reduce data sparsity and limit the influence of very low-frequency diagnostic categories on subsequent analyses (**Supplementary Figure S1**).

ASD diagnoses (F84.x) were used to identify the study population but were removed from the clustering variables to avoid defining groups based on the inclusion criterion.

### 2.4 Construction of patient-level diagnostic representations

For each patient, each diagnostic category was encoded as present (1) or absent (0), generating a binary patient-by-diagnosis matrix representing the presence of lifetime comorbidities during the study period. Repeated occurrences of the same diagnosis for a patient were counted only once.

Patients without any recorded comorbid diagnoses were excluded from the clustering procedure because their feature vectors contained no information for distinguishing between diagnostic profiles. These individuals were analyzed separately as a clinically relevant subgroup.

### 2.5 Self-Organizing map representation

A Self-Organizing Map (SOM) (14) was selected as the representation-learning component. The selection of SOM was motivated by both methodological and practical reasons, considering that the objective of this study was not only dimensionality reduction but also the generation of an interpretable structure that could be visualized, inspected by clinicians, and used as the basis for clustering. SOMs provide a topology-preserving projection of high-dimensional patient profiles into a two-dimensional space, unlike methods such as principal component analysis (PCA), which primarily summarize variance. Furthermore, SOMs generate an explicit grid-based representation whose prototypes can be directly inspected, visualized, and clustered. In contrast, methods such as t-SNE and UMAP are typically used to construct low-dimensional embeddings and do not inherently provide this additional topological structure.

The SOM was trained on the binary diagnostic matrix to organize patients according to similarities in their diagnostic profiles.

SOM hyperparameters were determined through sensitivity analysis evaluating the impact of training iterations, map size, learning rate, and random initialization on SOM performance and clustering robustness. Alternative configurations were compared using quantization error, topographic error, cluster stability, and profile reproducibility.

Based on these analysis a SOM consisting of a 20 × 20 hexagonal lattice with a Gaussian neighborhood function (σ = 2.0) and an initial learning rate of 0.5 was selected. Weights were initialized using randomly selected observations from the dataset. Training was performed in two consecutive stages of 10,000 iterations each using random sample selection. Euclidean distance was used during SOM training and identification of best-matching units (BMUs). Following training, each participant was assigned to its BMU within the resulting map.

SOM quality was evaluated using quantization error and topographic error, which assess representation accuracy and preservation of neighborhood relationships, respectively. Quantization error measures the average discrepancy between observations and their corresponding BMUs, whereas topographic error reflects the extent to which topological relationships are preserved within the reduced representation. To support interpretation of the diagnostic landscape, U-matrix visualizations and component-plane representations were generated. In addition, BMU density maps were inspected to evaluate participant distribution across the SOM space.

Because the diagnostic representation consisted of binary variables, additional sensitivity analysis were conducted to assess the impact of distance-metric selection on the resulting phenotypic structure. Alongside the Euclidean distance used in the primary analysis, Cosine, Manhattan, Chebyshev, Hamming, and Jaccard distances were evaluated. Solutions were compared using internal clustering validity metrics, phenotype-profile agreement, and patient-level agreement to determine whether the identified phenotypes were dependent on a specific distance formulation.

### 2.6 Identification of phenotype groups

Hierarchical agglomerative clustering was applied to SOM codebook vectors (neuron prototypes) rather than directly to individual patients. Clustering neuron prototypes allowed neighboring regions of the SOM with similar diagnostic profiles to be merged into larger and more interpretable phenotype groups.

Agglomerative clustering was performed using Ward linkage and Euclidean distance, as implemented in the clustering procedure. Each patient was subsequently assigned to the phenotype corresponding to the cluster membership of its BMU.

To identify an appropriate number of phenotypes, solutions were evaluated using multiple complementary criteria, including the silhouette coefficient, Dunn index, Calinski-Harabasz index, cluster-size distributions, and cluster balance ratio. Because different validity measures capture different aspects of clustering quality, no single metric was used as the sole selection criterion. Instead, final selection considered the combined evidence from internal validity metrics, preservation of clinically meaningful subgroup sizes, and avoidance of excessive fragmentation of the diagnostic landscape.

### 2.7 Internal validation and robustness assessment

Several analyses were conducted to evaluate the robustness of the proposed computational phenotyping workflow.

First, the influence of clustering methodology was assessed by comparing the SOM-based pipeline with hierarchical agglomerative clustering applied directly to the patient-level diagnostic matrix and with K-means clustering. Internal validity was evaluated using the silhouette coefficient, Dunn index, Calinski-Harabasz index, and cluster balance ratio. Agreement between resulting phenotypes was quantified through Hungarian matching of phenotype prevalence profiles and patient-level Adjusted Rand Index (ARI).

Second, sensitivity to distance-metric selection was evaluated by repeating the clustering procedure using Euclidean, Cosine, Manhattan, Chebyshev, Hamming, and Jaccard distances. Internal validity metrics, phenotype-profile agreement, and patient-level agreement were compared across solutions.

Third, bootstrap resampling was performed to evaluate phenotype reproducibility. For each bootstrap iteration, participants were resampled with replacement and the phenotype-generation workflow was repeated. Phenotype profiles were matched to the reference solution using Hungarian matching, and stability was quantified using mean, minimum, and maximum profile correlations.

These analyses complemented the SOM sensitivity analysis examining training iterations, grid size, learning rate, and random initialization.

### 2.8 Selection and characterization of phenotypes

Candidate phenotype solutions were evaluated using multiple internal validation metrics and cluster-structure characteristics. Specifically, silhouette coefficient, Dunn index, Calinski-Harabasz index, cluster-size distributions, and cluster balance ratio were examined across solutions ranging from K = 2 to K = 14.

Because different metrics favored different numbers of clusters, a compromise solution was selected. Preference was given to solutions maintaining clinically interpretable subgroup sizes while avoiding excessive fragmentation of the cohort. The final four-phenotype solution was retained because it provided meaningful differentiation of diagnostic profiles while preserving adequate subgroup sizes and maintaining an interpretable representation of the diagnostic landscape.

Phenotypes were characterized by calculating diagnostic-category prevalence within each group. SOM component-plane visualizations and phenotype-specific prevalence distributions were used to support interpretation of the resulting phenotypic profiles.

### 2.9 Clinical review of phenotype plausibility and interpretation

Following computational phenotype generation, the resulting diagnostic profiles, prevalence patterns, and SOM visualizations were independently reviewed by two clinicians from the adult autism unit, each with a minimum of five years of clinical experience treating autistic individuals. Reviewers independently evaluated the clinical plausibility and interpretability of the identified phenotypes based on the diagnostic prevalence profiles and SOM component-plane visualizations, and reached concordant conclusions regarding the plausibility and interpretability of the four phenotypes. The review was intended to assess clinical plausibility and interpretability of the resulting phenotype representations. It was not designed as a formal validation procedure and did not influence phenotype generation, clustering, or model selection.

## 3 Results

### 3.1 Cohort characteristics

A total of 927 adults with ASD from a real-world psychiatric EHR cohort were included in the study. Among them, 183 individuals (19.7%) had no recorded comorbid diagnosis beyond ASD and were therefore excluded from the computational phenotyping process. These individuals were retained as a separate descriptive subgroup.

The remaining 744 participants (80.3%) presented at least one comorbid diagnostic category and were included in the computational analysis. Number of diagnoses per participant are summarized in **Table 1**.

**Table 1:** Distribution of participants according to the number of recorded diagnostic categories.

	0	1	2	3	4	5	6	7	8	9	10
Original Diagnoses	177	232	195	109	109	66	20	10	5	3	1
Diagnoses Filtered	183	231	206	109	105	62	20	6	4	1	0

Autism spectrum disorder diagnoses used for cohort selection were excluded from the analysis. Diagnostic categories were aggregated according to ICD-10 block groupings and coded as present (1) or absent (0). Categories recorded in fewer than 1.5% of participants (n < 14) were excluded during preprocessing.

Diagnostic aggregation categories are presented in **Supplementary Table S1**. After diagnostic aggregation and frequency filtering, the final matrix included 11 diagnostic categories.

### 3.2 Diagnostic structure of the cohort

**Supplementary Figure 1** presents the frequency distribution of all aggregated diagnostic categories. **Table 2** reports only those categories meeting the inclusion criterion of at least 1.5% prevalence in the cohort (n ≥ 14). The most frequently observed categories were anxiety-related disorders (44.1%), mood disorders (27.6%), personality disorders (25.8%), developmental disorders (23.5%), intellectual disability (22.4%), and psychotic disorders (21.7%). Most participants presented multiple diagnostic categories, supporting the presence of substantial multimorbidity and the use of multivariate phenotyping approaches.

**Table 2:** Prevalence of diagnostic categories included in the computational phenotyping analysis.

	F10-19	F20-29	F30-39	F40-48	F50-59	F60-69	F70-79	F80-89	F90-98	F99	G40-47
N	109	201	256	409	33	239	208	26	218	98	106
%	11,8	21,7	27,6	44,1	3,6	25,8	22,4	2,8	23,5	10,6	11,4

### 3.3 Phenotype identification

Candidate solutions ranging from K = 2 to K = 14 were evaluated using multiple clustering-validity metrics and cluster-size characteristics. Different indices favored different clustering solutions (**Figure 1A**). Silhouette values increased progressively with larger numbers of clusters, whereas Calinski-Harabasz values favored more parsimonious solutions. Dunn index values stabilized after intermediate cluster numbers. Examination of cluster-size distributions showed progressive fragmentation of the cohort as the number of clusters increased, resulting in increasingly small subgroup sizes (**Figure 1B**).

**Figure 1:**
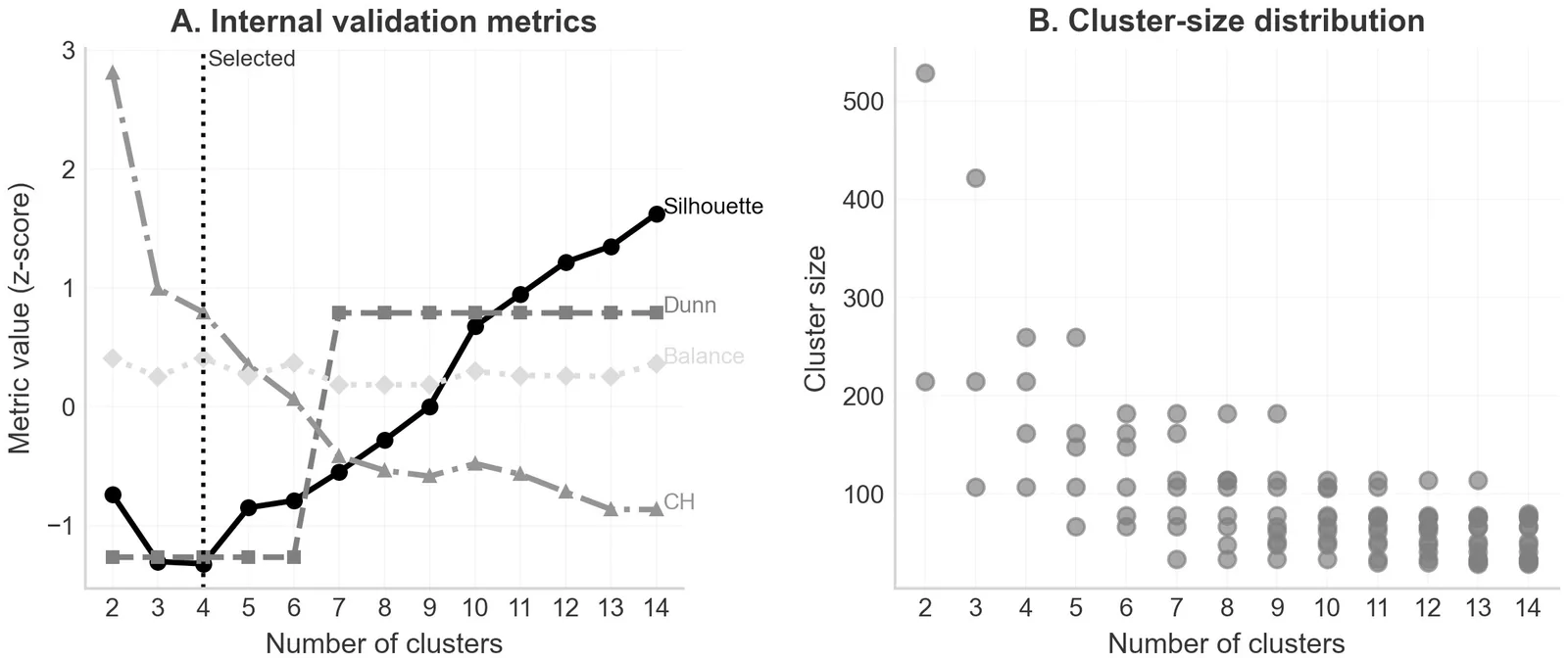
Cluster solution evaluation. **(A)** Internal validation metrics, **(B)** Cluster size distributions. The vertical dashed line indicated the selected four-phenotype solution.

Considering the combined evidence from internal validity metrics, cluster-size distributions, and interpretability of the resulting diagnostic structures, the four-phenotype solution was retained for subsequent analyses. Notably, all identified phenotypes contained more than 10% of the clustered cohort, whereas solutions with larger numbers of phenotypes increasingly generated small clusters with limited clinical relevance.

### 3.4 Internal validation and robustness

Comparison of the SOM-based workflow with conventional clustering approaches demonstrated similar clustering quality across methods (**Figure 2A**). Internal validation metrics showed only modest differences between SOM, hierarchical clustering, and K-means solutions. Phenotype-level agreement was high across methods (correlations 0.82-0.9), whereas patient-level agreement was lower (ARI 0.22-0.36; **Figure 2B**). These findings indicate that similar phenotypic structures emerged across alternative clustering approaches despite differences in individual patient assignments.

**Figure 2:**
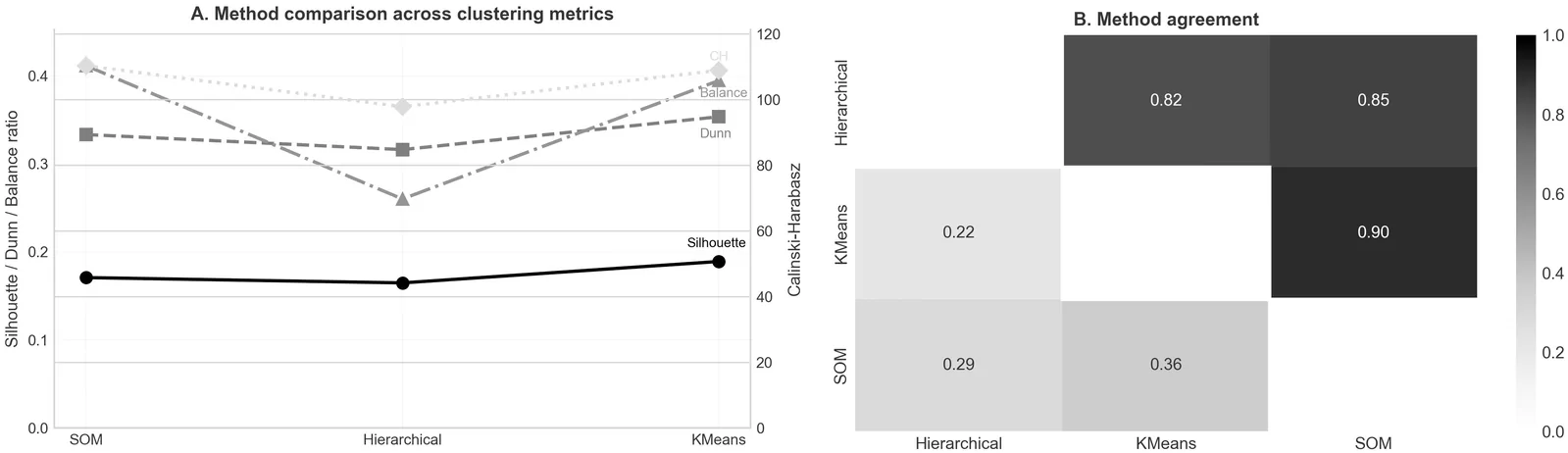
Comparison of clustering methods. **(A)** Method comparison across clustering metrics, **(B)** Agreement matrix showing phenotype-profile correlations (upper triangle) and patient-level Adjusted Rand Index values (lower triangle). Note. Methods compared include the SOM-based workflow, hierarchical clustering, and K-means clustering.

Distance-metric sensitivity analysis produced broadly similar clustering structures across Euclidean, Cosine, Manhattan, Hamming, and Jaccard formulations (**Supplementary Figure S2A**). Internal validity metrics showed relatively small differences across most distance measures, whereas Chebyshev distance produced a more distinct solution. Agreement between phenotype prevalence profiles remained high across distance formulations (correlations 0.79-0.98), although patient-level agreement was more variable (ARI 0.13-0.57; **Supplementary Figure S2B**).

Bootstrap resampling demonstrated substantial phenotype-level reproducibility (**Supplementary Figure S3**). Mean phenotype-profile correlations remained consistently high across bootstrap iterations, while maximum profile correlations approached unity in most resamples. Greater variability was observed for minimum profile correlations, suggesting that some phenotypes were reproduced more consistently than others.

### 3.5 Phenotype characterization

The identified phenotypes displayed distinct combinations of psychiatric and developmental comorbidity rather than differences driven by a single diagnostic category (**Figure 3**).

**Figure 3:**
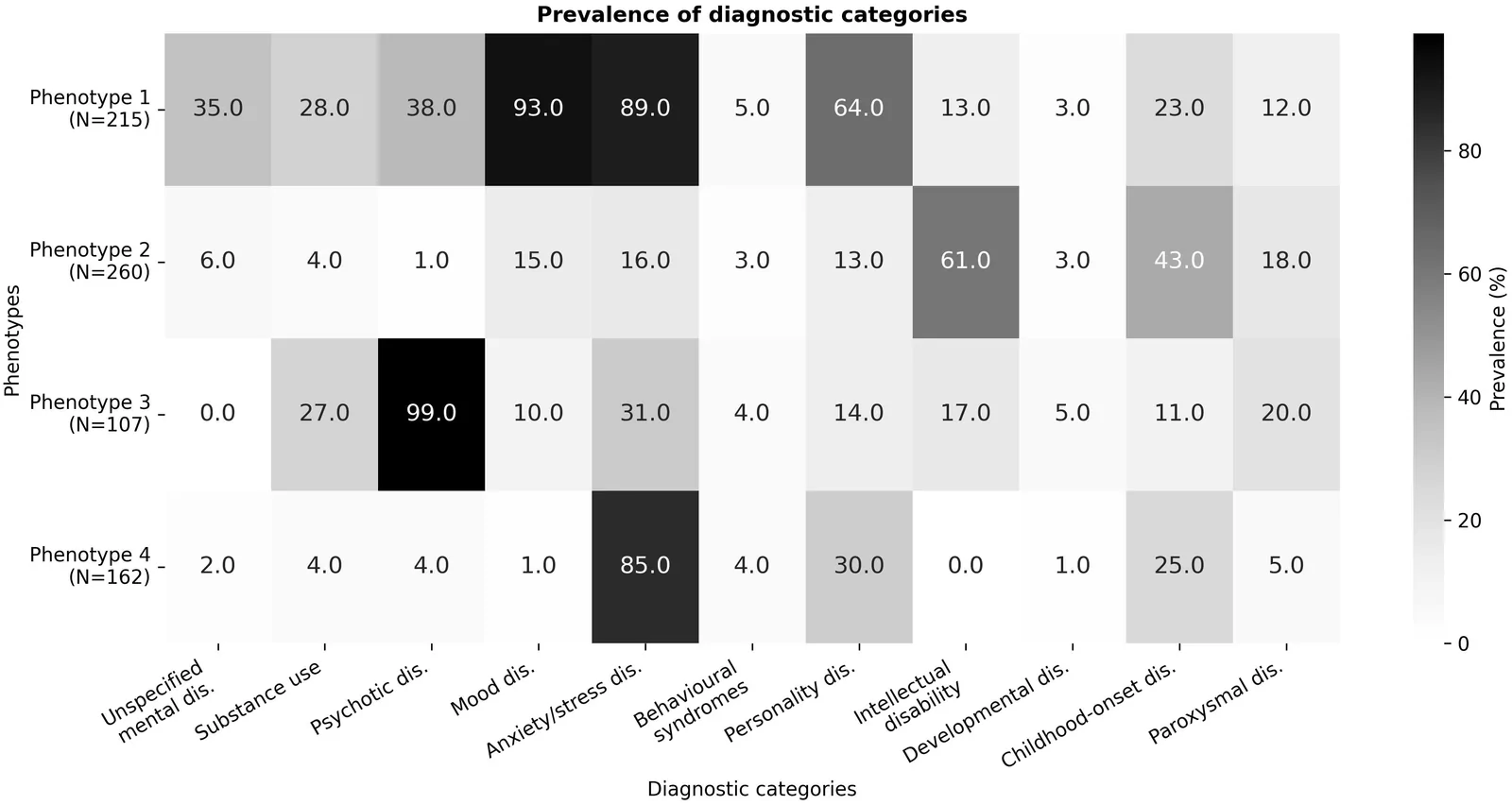
Prevalence of diagnostic categories across computational phenotypes. Values represent the percentage of participants within each phenotype presenting a given diagnostic category.

Phenotype 1 (n=215; 28.9%) was characterized by elevated prevalence of mood, anxiety, and personality-related disorders. Phenotype 2 (n=260; 34.9%) was characterized by developmental conditions and intellectual disability. Phenotype 3 (n=107; 14.4%) showed increased prevalence of psychotic and substance-related disorders. Phenotype 4 (n=162; 21.8%) was characterized primarily by anxiety and personality-related conditions. Phenotypes 1 and 4 shared a similarly high prevalence of anxiety-related disorders (89.0% and 85.0%), but diverged sharply in mood disorder prevalence (93.0% vs. 1.0%), with Phenotype 1 also showing higher personality-related, psychotic, and substance-related comorbidity.

Visualization of SOM component planes (**Supplementary Figure S4**) and phenotype prevalence profiles demonstrated clear differences in the contribution of diagnostic categories across phenotypes.

Participant distribution across the SOM space was assessed using a BMU density map (**Supplementary Figure S5**).

Independent review by two clinicians with expertise in adult autism concluded that the identified phenotypes were clinically plausible and interpretable representations of the observed diagnostic heterogeneity.

### 3.6 Sensitivity analysis and solution robustness

Sensitivity analysis were performed to evaluate the robustness of SOM parameter selection and phenotype identification.

Quantization error progressively decreased as the number of training iterations increased, while topographic error rapidly converged towards low values, indicating stabilization of the SOM representation after approximately 10,000 training iterations for each of the two rounds (**Supplementary Figure S6**).

Increasing SOM grid size improved representation accuracy, reducing both quantization and topographic errors. However, improvements became progressively smaller beyond the selected 20×20 configuration, supporting its use as a compromise between representation fidelity and model complexity (**Supplementary Figure S7**).

Learning-rate sensitivity analysis showed that a learning rate of 0.5 yielded the lowest quantization error among the evaluated values (**Supplementary Figure S8**).

Across 50 random initializations, patient-level cluster assignments showed moderate stability (ARI range 0.10–0.53). Nevertheless, phenotype-level diagnostic profiles exhibited substantially greater reproducibility, with profile correlations typically ranging between 0.6 and 1.0 (**Supplementary Figure S9**). This suggests that variability was primarily driven by reassignment of boundary cases rather than instability of the underlying phenotype structure.

Stability varied across diagnostic domains. Mood disorders, anxiety-related disorders, intellectual disability, and unspecified psychiatric diagnoses demonstrated the highest reproducibility, whereas low-prevalence categories such as neurodevelopmental and paroxysmal disorders showed substantially greater variability across repeated model initializations (**Supplementary Figure S10**).

The final SOM demonstrated low quantization error (0.183) and topographic error (0.013), indicating accurate representation of patient diagnostic profiles and preservation of neighborhood relationships.

## 4 Discussion

This study developed and evaluated an interpretable computational phenotyping workflow for routinely collected psychiatric EHR data. The proposed pipeline integrates clinically informed preprocessing, representation learning, clustering, visualization, and expert review to transform ICD-10 diagnostic information into structured and clinically interpretable structure of patient heterogeneity. Although phenotype groups were identified within the study cohort, the primary objective was to evaluate whether routine diagnostic data could be organized into stable, transparent, and clinically reviewable structures suitable for exploratory phenotyping and future clinical interpretation.

Previous EHR phenotyping studies have employed a variety of approaches including hierarchical clustering, latent class methods, tensor factorization, NLP-driven phenotype extraction, and dimensionality-reduction techniques (2, 8). These approaches have demonstrated the value of EHRs for identifying clinically relevant patient subgroups but often provide limited insight into relationships between phenotypes or the structure of the underlying diagnostic landscape. In contrast, the present pipeline combines clustering with a topology-preserving representation that enables simultaneous phenotype identification and visual exploration of patient similarity patterns.

Previous work has demonstrated the feasibility of identifying ASD subgroups from routine EHR data, although differences in population, diagnostic scope, and methodology make direct comparisons challenging (18). In the present study, we focused on adults with ASD and employed a structured workflow based on clinically aggregated psychiatric and neurodevelopmental diagnoses to support phenotype identification and interpretation.

An additional contribution of the proposed approach is the use of SOMs to provide a structured representation of the diagnostic landscape. The SOM revealed gradual transitions between phenotype regions and facilitated visualization of diagnostic-category distributions through component-plane representations. This is particularly relevant in psychiatry, where patient heterogeneity frequently exists on continua rather than as clearly separated groups. The ability to inspect neighboring regions of the map may therefore provide information that would not be captured by cluster assignments alone. Although four phenotypes were identified in the present cohort, the clinical interpretation of these specific diagnostic patterns is secondary to the methodological contribution of the study, since the principal objective was to evaluate the feasibility and interpretability of the proposed workflow.

From a clinical perspective, the identified phenotypes should not be interpreted as definitive ASD subtypes. Instead, they represent clinically interpretable patterns of co-occurring conditions that may support future investigations into heterogeneity within autistic populations. Such representations could enable cohort stratification, hypothesis generation, characterization of service needs, and identification of patient groups with similar psychiatric profiles (1). Importantly, inclusion of expert clinical review as a final stage of the workflow was intended to ensure that computationally derived patterns remained interpretable and clinically plausible before being considered for research or service-planning applications (19). The robustness analysis suggest that the proposed approach performs more consistently for common diagnostic categories that contribute substantially to the overall diagnostic structure. In contrast, lower-prevalence conditions displayed greater variability across model initializations, indicating that larger datasets or complementary clinical information may be required when the objective is to characterize rare diagnostic profiles.

An important aspect of the proposed workflow is that the resulting representations were considered interpretable and clinically plausible by clinicians with expertise in adult autism. This assessment should be interpreted as an expert plausibility review rather than as a formal external validation procedure. Nevertheless, the review provides preliminary evidence that the identified phenotype structures are understandable from a clinical perspective and may be useful for exploratory investigation of patient heterogeneity and hypothesis generation.

Several limitations should be considered. First, the study was conducted in a single healthcare setting, limiting the ability to evaluate generalizability. Second, routine diagnostic information is influenced by documentation practices and may not fully capture the complete clinical history of an individual (1). Third, methodological decisions related to diagnostic aggregation, feature selection, and phenotype identification may influence the final representations. Finally, the present analysis relied exclusively on diagnostic information and did not incorporate symptom severity, treatment response, functional outcomes, or longitudinal trajectories.

Although demonstrated in autistic adults, the workflow was designed around characteristics frequently observed across psychiatric EHR data, including multimorbidity, diagnostic sparsity, and heterogeneous clinical presentations. Future studies should evaluate whether similar computational phenotyping strategies can be applied successfully to other psychiatric and neurodevelopmental populations and across different healthcare systems.

## 5 Conclusions

This study presents an end-to-end computational phenotyping pipeline for routinely collected psychiatric EHR data. The proposed workflow successfully transformed sparse and heterogeneous ICD-10 diagnostic information into stable and interpretable patient profiles, enabling visualization of the diagnostic landscape and identification of coherent phenotype groups. Application of the pipeline in a real-world cohort of adults with ASD demonstrated the feasibility of deriving meaningful phenotypic representations from routine clinical data.

Beyond phenotype identification, the results highlight the value of combining representation learning, clustering, and visualization within a single interpretable framework. The SOM-based approach facilitated exploration of relationships between patient profiles and supported clinical review of the resulting structures. Robustness analysis further suggested that the workflow performs more consistently for common diagnostic categories, whereas less prevalent diagnoses may require larger samples or additional sources of clinical information to achieve stable characterization. Importantly, the resulting representations were considered clinically plausible and useful for exploring patient heterogeneity by clinicians with expertise in adult autism, supporting the practical interpretability of the proposed workflow.

Future work should evaluate the reproducibility of the pipeline across different healthcare settings and independent patient cohorts, as well as its applicability to other psychiatric and neurodevelopmental populations. Additional studies should also explore the integration of complementary clinical information beyond diagnoses, including symptom severity, functional outcomes, treatment history, and longitudinal trajectories. Finally, further expert-led investigation of the identified patterns will be necessary to extend interpretation beyond computational similarity and assess their potential clinical relevance.

## Data Availability

The datasets presented in this article are not readily available because the data supporting the findings of this study are not publicly available due to ethical, legal, and institutional restrictions related to the use of clinical EHRs. Requests for access to de-identified data may be considered by the authors upon reasonable request and subject to approval by the appropriate institutional, ethical, and legal review processes. Access cannot be guaranteed and will depend on compliance with applicable data protection regulations and institutional policies. Requests to access the datasets should be directed to jordi.rodeiro@sjd.es.
