# Supplementary material for: An interpretable computational phenotyping pipeline for adult autism using routine electronic health records
Source: Front Psychiatry. 2026 Sep 15;17:1952612. doi: 10.3389/fpsyt.2026.1952612 (PMC13619622; doi:10.3389/fpsyt.2026.1952612)
Supplement: Supplementary file 1 [file SupplementaryFile1.docx]

Supplementary Material

# 1 Supplementary Tables

**Supplementary Table S1.** Diagnostic Categories Considered During Preprocessing and Inclusion Criteria. *Note*. Diagnoses from ICD-10 Chapters F, G, and Q were considered for inclusion in the computational phenotyping analysis. Diagnostic categories were defined using ICD-10 block-level groupings. Categories with a prevalence below 1.5% of the cohort (n < 14) were excluded during preprocessing to reduce sparsity and improve the stability and interpretability of the resulting phenotypic representations.

| ICD-10 group | Description of the group |
| --- | --- |
| F01–F09 | Organic, including symptomatic, mental disorders |
| F10–F19 | Mental and behavioural disorders due to psychoactive substance use |
| F20–F29 | Schizophrenia, schizotypal and delusional disorders |
| F30–F39 | Mood (affective) disorders |
| F40–F48 | Neurotic, stress-related and somatoform disorders |
| F50–F59 | Behavioural syndromes associated with physiological disturbances and physical factors |
| F60–F69 | Disorders of adult personality and behaviour |
| F70–F79 | Intellectual disabilities |
| F80–F89 | Disorders of psychological development |
| F90–F98 | Behavioural and emotional disorders with onset usually occurring in childhood and adolescence |
| F99 | Unspecified mental disorder |
| G00–G09 | Inflammatory diseases of the central nervous system |
| G10–G14 | Systemic atrophies primarily affecting the central nervous system |
| G20–G26 | Extrapyramidal and movement disorders |
| G30–G32 | Other degenerative diseases of the nervous system |
| G35–G37 | Demyelinating diseases of the central nervous system |
| G40–G47 | Episodic and paroxysmal disorders |
| G50–G59 | Nerve, nerve root and plexus disorders |
| G60–G65 | Polyneuropathies and other disorders of the peripheral nervous system |
| G70–G73 | Diseases of myoneural junction and muscle |
| G80–G83 | Cerebral palsy and other paralytic syndromes |
| G89–G99 | Other disorders of the nervous system |
| Q00–Q07 | Congenital malformations of the nervous system |
| Q10–Q18 | Congenital malformations of eye, ear, face and neck |
| Q20–Q28 | Congenital malformations of the circulatory system |
| Q30–Q34 | Congenital malformations of the respiratory system |
| Q35–Q37 | Cleft lip and cleft palate |
| Q38–Q45 | Other congenital malformations of the digestive system |
| Q50–Q56 | Congenital malformations of genital organs |
| Q60–Q64 | Congenital malformations of the urinary system |
| Q65–Q79 | Congenital malformations and deformities of the musculoskeletal system |
| Q80–Q89 | Other congenital malformations |
| Q90–Q99 | Chromosomal abnormalities, not elsewhere classified |

# 2 Supplementary Figures

**Supplementary Figure S1.** **Frequency Distribution of Diagnostic Categories Prior to Filtering.** The dashed line indicates the minimum inclusion threshold corresponding to 1.5% of the study population (14 patients). Diagnostic categories below this threshold were excluded from the computational phenotyping analysis.

**Supplementary Figure S2.** **Distance-metric sensitivity analysis.** (A) Distance comparison across clustering metrics, (B) Agreement matrix showing phenotype-profile correlations (upper triangle) and patient-level Adjusted Rand Index values (lower triangle).

**Supplementary Figure S3.** **Bootstrap phenotype stability.** (A) Mean profile correlations across bootstrap iterations, (B) Minimum profile correlations across bootstrap iterations, (C) Maximum profile correlations across bootstrap iterations. Note. Phenotype profiles from bootstrap samples were matched to the reference solution using Hungarian matching.

**Supplementary Figure S4.** **Component-Plane Representations of Diagnostic Categories.** Component planes showing the distribution of each diagnostic category across the SOM. Higher values indicate a greater contribution of the corresponding diagnostic category to specific regions of the diagnostic space.

**Supplementary Figure S5.** **Best-Matching Unit (BMU) Density Map**. (A) Quantization error, (B) Topographic error. Note. Color intensity indicates the number of participants assigned to each neuron.

**Supplementary Figure S6.** Sensitivity of SOM performance to training iterations. (A) Quantization error, (B) Topographic error. Quantization error and topographic error decreased progressively with increasing training iterations and stabilized after approximately 10,000 epochs for each training round.

**Supplementary Figure S7.** **Effect of SOM grid size on representation accuracy.** (A) Quantization error, (B) Topographic error. Larger grids reduced quantization and topographic errors, although improvements diminished beyond the selected 20×20 SOM.

**Supplementary Figure S8.** **Learning-rate sensitivity analysis.** The selected learning rate (0.5) minimized quantization error among the evaluated configurations.

**Supplementary Figure S9.** **Stability of clustering solutions.** (A) Patient assignment stability, (B) Phenotype profile stability. Patient-level assignment stability was moderate (ARI), whereas phenotype-level diagnostic profiles showed substantially higher reproducibility (profile correlation). Note: Stability tested for 50 random SOM initializations.

**Supplementary Figure S10.** **Diagnosis stability.** Reproducibility of phenotype-defining diagnostic domains across random initializations. Values represent mean correlations of diagnostic prevalence patterns between matched phenotype solutions.
